# Developing forecasting model for future pandemic applications based on COVID-19 data 2020–2022

**DOI:** 10.1371/journal.pone.0285407

**Published:** 2023-05-12

**Authors:** Wan Imanul Aisyah Wan Mohamad Nawi, Abdul Aziz K. Abdul Hamid, Muhamad Safiih Lola, Syerrina Zakaria, Elayaraja Aruchunan, R. U. Gobithaasan, Nurul Hila Zainuddin, Wan Azani Mustafa, Mohd Lazim Abdullah, Nor Aieni Mokhtar, Mohd Tajuddin Abdullah

**Affiliations:** 1 Faculty of Ocean Engineering Technology and Informatics, Universiti Malaysia Terengganu, Kuala Nerus, Terengganu, Malaysia; 2 Special Interest Group on Applied Informatics and Intelligent Applications (AINIA) Universiti Malaysia Terengganu, Kuala Nerus, Terengganu, Malaysia; 3 Special Interest Group on Modeling and Data Analytics (SIGMDA), Universiti Malaysia Terengganu, Kuala Nerus, Terengganu, Malaysia; 4 Faculty of Science, Institute of Mathematical Sciences, Universiti Malaya, Kuala Lumpur, Kuala Lumpur Federal Territory, Malaysia; 5 Mathematics Department, Faculty of Science and Mathematics, Universiti Pendidikan Sultan Idris, Tanjong Malim, Perak Darul Ridzuan, Malaysia; 6 Faculty of Electrical Engineering & Technology, Universiti Malaysia Perlis, UniCITI Alam Campus, Sungai Chuchuh, Padang Besar, Perlis, Malaysia; 7 Advanced Computing (AdvCOMP), Centre of Excellence, Universiti Malaysia Perlis (UniMAP), Arau, Perlis, Malaysia; 8 Institute of Oceanography and Environment, Universiti Malaysia Terengganu, Kuala Nerus, Terengganu, Malaysia; 9 Faculty of Fisheries and Food Science, Universiti Malaysia Terengganu, Kuala Nerus, Terengganu, Malaysia; 10 Fellow Academy of Sciences Malaysia, Kuala Lumpur, Kuala Lumpur Federal Territory, Malaysia; King Abdulaziz University, SAUDI ARABIA

## Abstract

Improving forecasting particularly time series forecasting accuracy, efficiency and precisely become crucial for the authorities to forecast, monitor, and prevent the COVID-19 cases so that its spread can be controlled more effectively. However, the results obtained from prediction models are inaccurate, imprecise as well as inefficient due to linear and non-linear patterns exist in the data set, respectively. Therefore, to produce more accurate and efficient COVID-19 prediction value that is closer to the true COVID-19 value, a hybrid approach has been implemented. Thus, aims of this study is (1) to propose a hybrid ARIMA-SVM model to produce better forecasting results. (2) to investigate in terms of the performance of the proposed models and percentage improvement against ARIMA and SVM models. statistical measurements such as MSE, RMSE, MAE, and MAPE then conducted to verify that the proposed models are better than ARIMA and SVM models. Empirical results with three real datasets of well-known cases of COVID-19 in Malaysia show that, compared to the ARIMA and SVM models, the proposed model generates the smallest MSE, RMSE, MAE and MAPE values for the training and testing datasets, means that the predicted value from the proposed model is closer to the actual value. These results prove that the proposed model can generate estimated values more accurately and efficiently. As compared to ARIMA and SVM, our proposed models perform much better in terms of error reduction percentages for all datasets. This is demonstrated by the maximum scores of 73.12%, 74.6%, 90.38%, and 68.99% in the MAE, MAPE, MSE, and RMSE, respectively. Therefore, the proposed model can be the best and effective way to improve prediction performance with a higher level of accuracy and efficiency in predicting cases of COVID-19.

## Introduction

The city of Wuhan in the province of Hubei, China is etched in the folds of history for being the first place of the spread of the Coronavirus disease (COVID-19), due to severe acute respiratory syndrome. The World Health Organisation (WHO) on 31^st^ January was firstly declared that COVID-19 as a “Public Health Emergency of International Concern” [1]. Originally, it was thought that the virus has been derived from a seafood market in Wuhan. However, on 11 January 2020 the genetic sequence of which was overtly shared by China through human-to-human contacts have driven its rapid spread with a total of 9,129,146 confirmed cases, including 473,797 deaths across the globe until June 24, 2020 [2]. Nonetheless, the COVID-19 pandemic has infected more than 151 million of the humans all over the world and caused 3 million deaths as of May 1, 2021. The countries like USA, Brazil, Russia, Spain, UK, Italy, France, Germany, China, India, Iran, and Pakistan become the most affected from COVID-19. The first few COVID-19 cases were reported in Malaysia on 24^th^ January 2020 were detected from Chinese tourists entering the country from Singapore [3]. In the early stage, only in single digit of daily cases were reported, however it had increased to 235 by 26^th^ March [4]. The number of daily cases in Malaysia were continued to rise exponentially hitting around 20,000 by August 2021. The Malaysian government was declared the implementation of the Movement Control Order (MCO), Conditional MCO (CMCO) and Recovery MCO (RMCO) from 18^th^ March to 12^th^ May 2020, 13^th^ May to 9^th^ June, and 9^th^ June to 31^st^ December, respectively. All travelling and socio-economic activities (gatherings for religious and cultural occasions were not allowed) were restricted nationwide to keep new infections at bay and avoid overloading the country’s healthcare system during this period. All government and private offices, and education institutions including transport hubs were closed and instructing citizens to stay at home and interstate travelling was banned with fines of up to RM10,000 for violators.

Since WHO declared as the outbreak of COVID-19 as a pandemic, a lot of effort have been attempts not only from government worldwide but effort also from medical institution are committed to finding vaccines and treatments to control the spread of the virus, statistical modelling particularly forecasting on the COVID-19 cases also have been extensively carried out by statisticians and health scientists to support the health system to inhibit the disaster of infection as well. In this scenario, the capability to pinpoint the growth rate more effectively at which the epidemic is spreading is very crucial to fight back and assist the governments mindfulness concerning society planning and policymaking to accurately deal with the consequences of the infection. Thus, the motivation behind this research compared to the existing research work, namely, (i) to develop the forecasting model that more accurate and efficient regarding the spread of COVID-19 in Malaysia, and (ii) to compare the performance of this novel model with ARIMAS and SVM. This model can assist the public health authorities for pre-emptive and preventive planning to curtail the impact of future pandemics.

During pandemic many studies have been carried out through different mathematical and statistical models to predict the spread of the COVID-19 pandemic. One of the most popular and widely time series forecasting models used to analyse and predict the spread of the disease is the ARIMA (*p*,*d*, *q*) model [5–7]. Forecasting daily new cases of COVID-19 was a difficult undertaking because the cases were growing daily. In the first wave, the cases of COVID-19 pattern has been continuously increasing for some period then decline. However, for the second wave it seen to be increased again and some of the COVID-19 cases are difficult to predict. In this scenario, a few researchers predict COVID-19 pattern using ARIMA [8–15]. However, ARIMA model have a limitation where it’s normally only can handle a linear time series data structure [16]. However, approximations by ARIMA models are inadequate in representing a barrier in time series forecasting for researchers particularly for nonlinear pattern [17]. Despite its superior performance, Support Vector Machines (SVM’s) classification performance and classifier’s generalisation ability are frequently impacted by the dimension or quantity of feature variables as mentioned by Lee [18] is used. As a sequence of the development of Vector Machines model, this process will be able to provide the accurate and efficient result in any case of prediction. The SVMs, which were first introduced by Vladimir Vapnik in 1995 [19] in the domain of statistical learning theory and structural risk minimization, have been shown to operate well on a variety of forecasting and classification issues. The SVMs could also cope with or address difficulties like nonlinearity, local minimum, and high dimension in which ARIMA model [16, 20–22]. SVMs models have recently been used to handle issues such as nonlinear, local minimum, and high dimension. SVMs can ensure higher accuracy for a long-term prediction compared to other computational approaches even in many practical applications. However, single SVM model as single ARIMA model also have some limitation where SVM model only can handle nonlinear data, instead of linear data. With the constrains of a single ARIMA and SVM models as well, in-dept analysis of time series forecasting, hybrid approaches become the best approach to overcome both limitations and it’s a very significant impact in numerous fields due to their dynamic nature and capability to predict at a higher level of accuracy, efficiency, and precision. This approach is crucial due to issues that arise in time series forecasting where almost all real-world time series contain both linear and nonlinear correlation patterns between the data. Recently, the hybridization of forecasting methods has been used with great achievement to reach enhanced forecasting accuracy [16, 17, 20–26].

In terms the spread of COVID-19, the hybrid time series model approach is crucial in predicting the impact of COVID-19 outbreak and it has been shown to be successful in predicting COVID-19 [27–30]. Thus. this study aims (a) to propose the hybrid ARIMA -SVM models approach for produce better forecasting results where its capability to produce the best estimator, i.e., generating small error terms; (b) to investigate the performance of the proposed models by comparing with the ARIMA and SVM models using three daily cases of COVID-19 data in Malaysia which are daily new positive cases, daily new fatalities cases, and daily new recovered cases. In spite of recent advances in time series and in particular in COVID-19, the model building process does not include cases of COVID-19 specifically in Malaysia to assist the authorities in dealing with the spread of this outbreak by producing more efficient, accurate and precise forecast results in the future. Therefore, in this study rather than rely on conventional approaches to deal with the COVID-19 data, this study relies on intelligent-based prediction methods to better predict the future pandemic. According to Moore [31], the scenario for the next likely new pandemic of strain of bird influenza H7N9 virus, or a novel coronavirus. Despite the fact that future outbreaks are inevitable, however, this intelligent-based prediction methods can produce more efficient, accurate and precise forecasts for pre-emptive prevention medicinal procedures by the local health care authorities [32, 33]. The model can also be used to predict Coronavirus or bird flu in the future, especially in tropical rainforest countries like Malaysia. Additionally, the intelligent-based prediction methods will produce prediction models that are more accurate, precise, and efficient in predicting the dynamic spread of the virus in the future. Although, the vaccine is currently available and the number of deaths worldwide is low, this model will be useful for making very accurate predictions if similar outbreaks occur in the future. As a result, the spread of COVID-19 can be predicted earlier so that better health facilities can be built, legislative measures can be taken, and economic losses, especially human losses, can be avoided.

The rest of this paper is organized as follows. Details of the method we used to develop our proposed model are discussed in materials and methods. Followed by a brief formulation of the hybrid ARIMA-SVM model used in this study. The performance of our proposed model based on three well-known COVID-19 case datasets is presented in the results and discussion. Finally, we conclude the paper and provide recommendations for further work.

### Materials and methods

#### The ARIMA modelling

The Autoregressive Integrated Moving Average, The ARIMA (*p*,*d*,*q*) model is one of the families in time series forecasting that is commonly used for time series forecasting because of its flexibility with various categories of time series datasets [17]. It also expressly caters to a set of standard patterns in time series analysis, enabling an easy-to-use yet powerful way for creating accurate time series predictions However, limitations may occur with pre-assumptions due to the existence of a linear form that is a linear relationship between the future value of the time series with the current value, past and white noise in the model [16–18, 22, 34]. In the ARIMA model, let *p* and *q* be the numbers of autoregressive and moving average terms and they are always mentioned in the order of the model while, *d* be the integer representative of the differential order. The type of ARIMA model with mean, *μ* is represented mathematically as follows.

$$y_{t}=\mu +\emptyset_{1}y_{t-1}+\emptyset_{2}y_{t-2}+\cdots +\emptyset_{p}y_{t-p}+\varepsilon_{t}-\theta_{1}\varepsilon_{t-1}-\theta_{2}\varepsilon_{t-2}-\cdots -\theta_{q}\varepsilon_{t-q}$$ (1)

where, *y*_*t*_ and *ε*_*t*_ are the actual value and the random error at time *t*, respectively. Both are assumed to be independently and identically distributed (*iid*) with mean 0 and constant variance of *σ*^2^, ∅_*i*_(*i* = 1,2,…,*q*) and *θ*_*j*_(*j* = 0,1,2,…,*q*) are the model parameters that need to be predicted.

### Support vector machines model

The support vector machine (SVM) introduced by Vladimir Vapnik [19] which involves statistical learning theory can better handle larger dimensional data, even with a small number of training examples, and has excellent generalization. Because the models choose limit support vectors from input data, they process data quickly. The SVM regression function is written as follows.

For linear and regressive data set {*x*_*i*_, *y*_*i*_} the function is formulated as follows

$$f(x)=w^{T}x+b$$ (2)

The coefficient *w* and *b* are estimated by minimizing

$$\frac{1}{2}w^{T}w+C\frac{1}{n}\sum_{i-1}^{n}L_{\varepsilon }(y_{i},f(x_{i}))$$ (3)

where *L_ε_* is called the *ε*-intensive loss function and is formulated as follows:

$$L_{\varepsilon }(y,f(x))=\left\{ \begin{array}{l} 0\;\mathrm{if}\;|y-f(x)|\leq \varepsilon \\ |y-f(x)|\;others \end{array}\right.$$ (4)

By introducing positive slack variable *ξ* and $\xi_{i}^{*}$, Eq (3) can be transformed to the following constrained formulation:

$$\begin{array}{l} min\frac{1}{2}w^{T}w+C\sum_{i=1}^{n}\left(\xi_{i}+\xi_{i}^{*}\right)\\ wx_{i}+b_{i}-y_{i}\leq \xi +\xi_{i}^{*}\\ -wx_{i}-b_{i}+y_{i}\leq \xi +\xi_{i}^{*}\\ \xi_{i},\xi_{i}^{*}\geq 0\\ i=1,2,\ldots ,N \end{array}$$ (5)

When solving the above formula, we always utilize dual theory to convert it into a convex quadratic programming problem. Introducing the Lagrange Eq(5) change into the following term:

$$min\frac{1}{2}\sum_{i,j=1}^{n}\left(\alpha_{i}^{*}-\alpha_{i})(\alpha_{j}^{*}-\alpha_{j})\alpha_{i}^{T}\alpha_{j}-\sum_{i=1}^{n}\alpha_{i}^{*}(y_{i}-\varepsilon )-\alpha_{i}(y_{i}+\varepsilon \right)$$ (6)

subject to

$$\sum_{i=1}^{n}\left(\alpha_{i}-\alpha_{i}^{*})=0,\;\alpha_{i},\alpha_{i}^{*}\in [0,C\right]$$

When the data set cannot be regressed linearly, we also map them to a high dimension feature space and make linear regress. Then the formulation is as follows:

$$min\frac{1}{2}\sum_{i,j=1}^{n}\left(\alpha_{i}^{*}-\alpha_{i})(\alpha_{j}^{*}-\alpha_{j})\varphi ({x_{i})}^{T}\varphi (x_{j})-\sum_{i=1}^{n}\alpha_{i}^{*}(y_{i}-\varepsilon )-\alpha_{i}(y_{i}+\varepsilon \right)$$ (7)

subject to

$$\sum_{i=1}^{n}\left(\alpha_{i}-\alpha_{i}^{*})=0,\alpha_{i},\alpha_{i}^{*}\in [0,C\right]$$

Let $K(X_{i},X_{j})=\{\varphi (X_{i})∙\varphi (X_{j})\}=\varphi^{T}(X_{j})\varphi {(X}_{i});\;K(x,x)$ is the inner product of feature space and is called kernel function. Any symmetric function that satisfies Mercer condition can be used as Kernel Function [19]. The Gaussian kernel function is specified in this study.


$$K(x_{i},x_{j})=exp(-||x_{i}-x_{i}{||}^{2}/(2\sigma^{2}))$$ (8)

The SVMs were employed to estimate the nonlinear behaviour of the forecasting data set as Gaussian kernels tend to give good performance under general smoothness assumptions [23].

### Proposed hybrid models

Despite various time series models presented, the accuracy, effectively as well as precisely of time series forecasting at this time become the fundamental to many decision-making processes. However, those factors do not occur in the ARIMA and SVM models. This also become the most reason why time series forecasting model is crucial, most challenging, and dynamic as well as active research in many fields of studies. ARIMA and SVM models also have achieved success in their linear or nonlinear areas [16, 25, 26]. However, none of these are generic principles that can be generalized to all situations. Hence, a hybrid strategy that employs both linear and nonlinear modelling skills is recommended. This approach is suggested mainly for improving overall prediction effectiveness. Therefore, there is no research on how to improve the effectiveness of forecasting models conducted especially in the case of COVID-19 in Malaysia.

In this study two motivation for hybrid models. First, a single model of ARIMA and SVM may not be sufficient to identify all the characteristics of the time series. Second, the assumption that either one or both cannot recognize the actual data generating process. Building the hybrid models of this study involved of two parts. Part I about linear autocorrelation composition and follow with nonlinear component in part II. Thus,

$$y_{t}=L_{t}+N_{t}$$ (9)

Where *L*_*t*_ and *N*_*t*_ signifies the linear composition and the nonlinear component, respectively. These two parts must be approximated based on the data. In the part I, linear modelling become the focus using ARIMA model to model the linear composition. The model from the first model involved the residuals which is the nonlinear interactions, and it cannot be model by linear model, and maybe linear relationship as well. Thus,

$$L_{t}=[\sum_{i=1}^{p}\emptyset_{i}z_{t-i}-\sum_{i=1}^{p}\theta_{j}\varepsilon_{t-j}]+e_{t}={\hat{L}}_{t}+e_{t}$$ (10)

Let *e*_*i*_ signify the residual from the linear model at time *t*, then

$$e_{t}=y_{t}-{\hat{L}}_{t}$$

where ${\hat{L}}_{t}$ is the predicted value for time *t* from the estimated relationship in (1) with *e*_*t*_ is the residual at time *t* from the linear model. According to Aisyah, et al., [16] the residual data set after ARIMA fitting will only contain non-linear relationships and can be properly represented by a linear model. Results of first stage which contains the forecast values and residuals of linear modelling then used in Part II.

In Part II, the focus is for nonlinear modelling which SVM used to model the nonlinear (maybe linear) relationship occurring in residuals of linear modelling and original data as well. Then, the residual can be calculated using SVM by modelling various configurations as follows:

$$e_{t}=f(e_{t-1},e_{t-2},..e_{t-n})+\varepsilon_{t}$$ (11)

$$e_{t}=f(e_{t-1},e_{t-12})+\varepsilon_{t}$$ (12)

$$y_{t}=f(y_{t-1},y_{t-12},\;{\hat{L}}_{t})+\varepsilon_{t}$$ (13)

$$y_{t}=f(y_{t-1},y_{t-12})+\varepsilon_{t}$$ (14)

where *f* is a nonlinear function determined by the SVMs model and *ε*_*t*_ is the random errors.

Thus, the combined forecast is

$${\hat{y}}_{t}={\hat{L}}_{t}+{\hat{N}}_{t}$$ (15)

**Fig 1:**
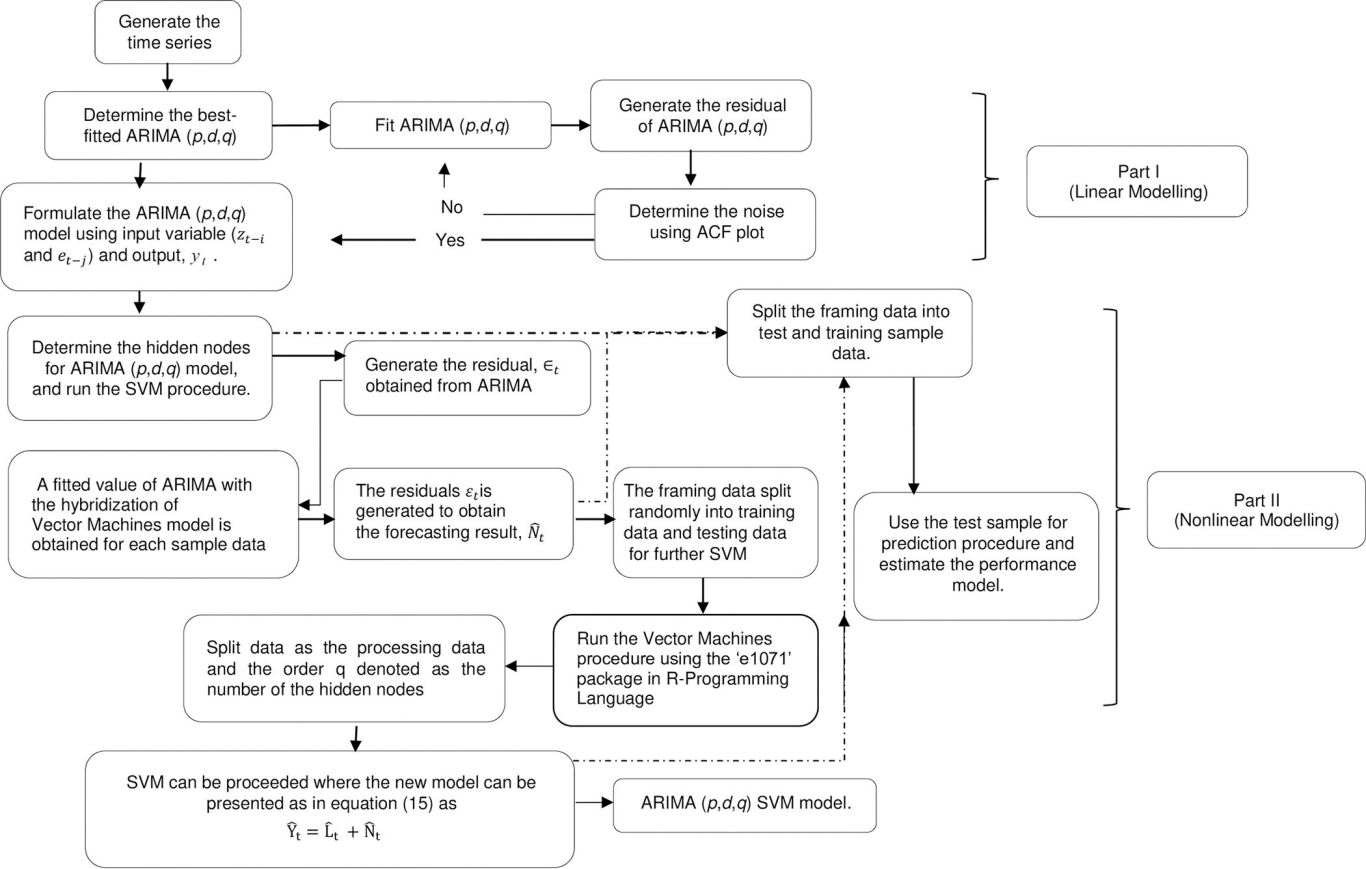
Flowchart process for hybrid ARIMA SVM models.

Eqs (11) and (12) can be identified as ${\hat{N}}_{t}$, therefore the forecasted values can be achieved by summation of linear and nonlinear components Fig 1 shows the functional flowchart of hybrid models

In short, the proposed methodology of the hybrid process consists of two parts. In the part I, the ARIMA model is employed to analyse the problem of linear composition. In the part II, a SVM model is developed to model the residuals from part I. Since the ARIMA model in part I cannot handle the nonlinear component of the data, the residuals of linear model will include information about the nonlinearity. The results from the SVM can be treated as forecasts of the error terms for the ARIMA model. The hybrid model utilizes the distinctive feature and strength of ARIMA and SVM model as well in defining various patterns. Therefore, it is more effective to model linear and non-linear patterns separately by using two different models and re-hybridize the forecast results obtained to improve overall modelling and forecasting performance.

### Proposed algorithm

**Step 1**: Three selected time series of COVID-19 cases datasets (1^st^ of October 2020-4^th^ of November 2022), namely daily new positive cases, daily new deaths cases and daily new recovered cases are generated in R programming Language

**Step 2**: Every of the generated datasets is defined as $\left\{X_{1i}=x_{11,}x_{12},x_{13,}\ldots ,x_{n1}\right\}$, $\left\{X_{2i}=x_{21,}x_{22},x_{23,}\ldots ,x_{2n}\right\}$ and $\left\{X_{3i}=x_{31,}x_{32},x_{33,}\ldots ,x_{3n}\right\}$ for daily new positive cases, daily new deaths cases and, daily new recovered cases, respectively. Then, selected the best ARIMA (*p*,*d*,*q*) after checking the autocorrelation function (ACF) plot of ARIMA (*p*,*d*,*q*) residuals. The best fitted value for daily new positive cases is ARIMA (2,1,2), while ARIMA (1,1,2) and ARIMA (0,1,1) for daily new fatalities cases, and daily new recovered cases of COVID-19, respectively.

**Step 3**: The fitted value, $y_{t-i}=(y_{t-1},y_{t-2},\ldots ,y_{t-m})$ and the residuals $e_{t-i}=(e_{t-1},e_{t-2},\ldots .,e_{t-n})$

**Step 4**: Combine the values in step 3 as a set of input variables to get the output *y*_*t*_

**Step 5**: The ARIMA (*p*,*d*,*q*) is defined by the order of *q*. According to the information in step 4, Vector Machines is carried out to examine the residuals to get the output *L*_*t*_ using R-programming Language.

**Step 6**: A fitted value of ARIMA with the hybridization of Vector Machines model is obtained for each sample data. Then, the residuals *ε*_*t*_ is generated to obtain the forecasting result, ${\hat{N}}_{t}$

**Step 7**: The framing data split randomly into training data and testing data for further Vector Machines model. Run the Vector Machines procedure using the ‘e1071’ package in R-Programming Language

**Step 8**: Assume the split data as the processing data and the order q as in Step 5. Therefore, the combine forecast as in Eq (15): ${\hat{Y}}_{t}={\hat{L}}_{t}+{\hat{N}}_{t}$

**Step 9**: Estimate the model performance using the statistical measurement which are MSE, RMSE, MAE and MAPE.

### Forecasting evaluation criteria

In order to evaluate the performance of the proposed hybrid models, the different statistical measurements criteria which followed by [16, 17, 32], such as MAE (Mean Absolute Error), MAPE (Mean Absolute Percentage Error), MSE (Mean Squared Error), and RMSE (Root Mean Squared Error) are used.


$$MAE=\frac{1}{n}\sum_{t=1}^{n}|\hat{y_{t}}-y_{t}|$$

$$MAPE=\frac{1}{n}\sum_{t=1}^{n}\left|\frac{\hat{y_{t}}-y_{t}}{y_{t}}\right|\times 100$$

$$MSE=\frac{1}{n}\sum_{t=1}^{n}{\left(\hat{y_{t}}-y_{t}\right)}^{2}$$

$$RMSE=\sqrt{\frac{1}{n}\sum_{t=1}^{n}{\left(\hat{y_{t}}-y_{t}\right)}^{2}}=\sqrt{MSE}$$

For ARIMA model, normally, the measurement tools such as Akaike’s information Criterion (AIC) and the Bayesian information criterion (BIC) have been widely used in time series analysis to determine the appropriate length for distributed lag [16, 17]. Therefore, model selection is made based on the model with the smallest value of AIC and BIC to provide measures of model performance which gives the selection of the best ARIMA model. Meanwhile, for the SVMs models, three parameters such as *γ*, *C* and *ε* are used as the measurement tools to determine the best fitted model. Inappropriate selection of SVM model parameters can result in either over or under fitting the training data. As with the ARIMA model, the parameter sets of the SVMs model with the lowest MSE value will be selected for use in the best fitting model. Thus, for the hybrid models, first the ARIMA worked as a pre-processor to filter the linear pattern of data sets. Then, the error term generated from the ARIMA model will be fed into the SVM in the hybrid models. The SVMs were performed to reduce the error function from the ARIMA.

## Results and discussion

### Application of the hybrid model to daily cases of COVID-19 in Malaysia

This section analysed the performance of the proposed model in respect to two aspects: (1) the performance of the proposed models against ARIMA and SVM models, and (2) the percentage improvement of the proposed models against ARIMA and SVM models. Since the World Health Organisation (WHO) was declared that COVID-19 is pandemic worldwide, the COVID-19 time series data sets have been widely studied. Next, the predictive capability of the developed novel models was compared using three well-known data sets of daily cases of COVID-19 in Malaysia- daily new positive cases data, daily new fatalities cases data and daily new recovered cases data–used to demonstrate the performance of the proposed model in terms of accuracy, effectively and accurately. All these data are reported from the 1^st^ of October 2020 to 4^th^ of November 2022 and retrieved from the COVIDNOW website at https://covidnow.moh.gov.my/

In the Table 1, the minimum value of the new death, new cases and new recovered are zero, 2600 and 1.8, respectively, while the maximum value of new cases, death and recovered cases are 33872.0, 592 and 33406 respectively. Similarly, the mean and median for the number of new cases, death and recovered cases are 6322.7, 47.51, 6415.5, where the parenthesis indicates the median in (3471, 11, 3447.0). While the first quartile value of daily new cases, death and recover cases are 1922, 4 and 1843 respectively. The third quartile value of number of daily new cases, death and recover cases are 6824, 58 and 6775 respectively. Moreover, the standard deviation of new cases, death and recover cases are 7097.8, 81.12 and 7058.3 respectively.

**Table 1:** Descriptive statistics of COVID-19 daily new cases, death and recovered cases of Malaysia.

	New Case	New Death	New Recovered
Min	2600	0	1.8
1^st^ Qu	1922	4	1843.0
Median	3471	11	3447.0
Mean	6415.5	47.5098	6322.7
3^rd^ Qu	6824	58	6775.0
Max	33406	592	33872.0
SD	7097.8	81.1215	7058.3

In addition, this section also discusses the process of proposed models at once for both part i.e., Part I (Linear modelling) and Part II (Nonlinear Modelling) using three well-known data sets of COVID-19 i.e., daily new positive cases, daily new deaths cases and daily new recovered cases are discussed in order to demonstrate the effectiveness of the proposal models. Both linear and nonlinear modelling as well as well as the data used in this study are executed through programming using the R-language.

**Part I (Linear Modelling)–**the best ARIMA model for the daily new positive case dataset is derived from ARIMA (2,1,2). The best fitting ARIMA model for the daily new death case data set is ARIMA (1,1,2). Meanwhile, in the case of the daily new recovered cases dataset, the best ARIMA model is reported as ARIMA (0,1,1). The results of this ARIMA (*p*,*d*,*q*) model are summarized in Table 2. The estimates of all parameters are shown in Table 3. From this table, it can be observed that the *p*-values of all parameters are small. Therefore, the models were statistically significant for confirmed, recovered, and death cases, and could be used to forecast the future [33, 35].

**Table 2:** The best ARIMA(*p*,*d*,*q*) model selection.

COVID-19 daily cases	ARIMA(*p*,*d*,*q*)	AIC	BIC
**Daily New Positive Cases**	(2,1,2)	12564.54	12587.73
**Daily New Deaths Cases**	(1,1,2)	6930.12	6948.63
**Daily New Recovered Cases**	(0,1,1)	13044.74	13054.01

**Table 3:** Parameter estimates of ARIMA (*p*,*d*,*q*) models and their *p*-values.

Model parameters	Estimate	z-stat	*p*-value
New Case *ARIMA(2*,*1*,*2)*			
*θ*_1_	1.2408	120.085	< 0.0001
*θ*_2_	-0.9715	-98.320	< 0.0001
*φ*_1_	-1.2628	-42.225	< 0.0001
*φ*_2_	0.8738	48.102	< 0.0001
Recovered Case *ARIMA(0*,*1*,*1)*			
*φ*_1_	-0.3473	-9.953	< 0.0001
Death Case *ARIMA(1*,*1*,*2)*			
*θ*_1_	0.8595	19.852	< 0.0001
*φ*_1_	-1.6196	-35.651	< 0.0001
*φ*_2_	0.7039	20.432	< 0.0001

**Part II (Nonlinear Modelling)–**In order to obtain an optimal machine learning algorithm, based on the concepts of support vector machine design and using pruning algorithms in R-programming software. For the daily new positive COVID-19 cases datasets, parameters γ = 2, C = 256, ε = 0.2 shows the smallest values of MSE i.e., 10321275 (see Table 4). Therefore, this parameters value was selected for use in the best-fitting model for the datasets of daily new positive COVID-19 cases. Whereas the smallest value of MSE is 1431.732 and 9885746 (Table 4), with parameters γ = 2, C = 256, ε = 0.2 are selected as the best-fitting model for daily new death cases of COVID-19 and daily new recovered cases of COVID-19, respectively.

**Table 4:** SVMs model parameters for the daily new COVID-19 cases datasets.

COVID-19 daily cases	SVM Parameter	MSE
**Daily New Positive Cases**	γ = 2, C = 128, ε = 0.1	11260319
	γ = 2, C = 8, ε = 0.2	17519221
	**γ = 2, C = 256, ε = 0.2**	**10321275**
	γ = 2, C = 4, ε = 0.3	19499587
	γ = 2, C = 128, ε = 0.3	11058873
**Daily New Deaths Cases**	γ = 2, C = 16, ε = 0.2	1599.659
	γ = 2, C = 128, ε = 0.2	1418.033
	γ = 2, C = 256, ε = 0.2	1378.962
	γ = 2, C = 8, ε = 0.3	1711.465
	**γ = 2, C = 256, ε = 0.3**	**1431.732**
**Daily New Recovered Cases**	γ = 0.5, C = 256, ε = 0.2	38847557
	γ = 1, C = 4, ε = 0.2	36617325
	γ = 1, C = 8, ε = 0.2	34666712
	γ = 2, C = 128, ε = 0.2	10463694
	**γ = 2, C = 256, ε = 0.2**	**9885746**

### New positive cases data forecasts

**Fig 2:**
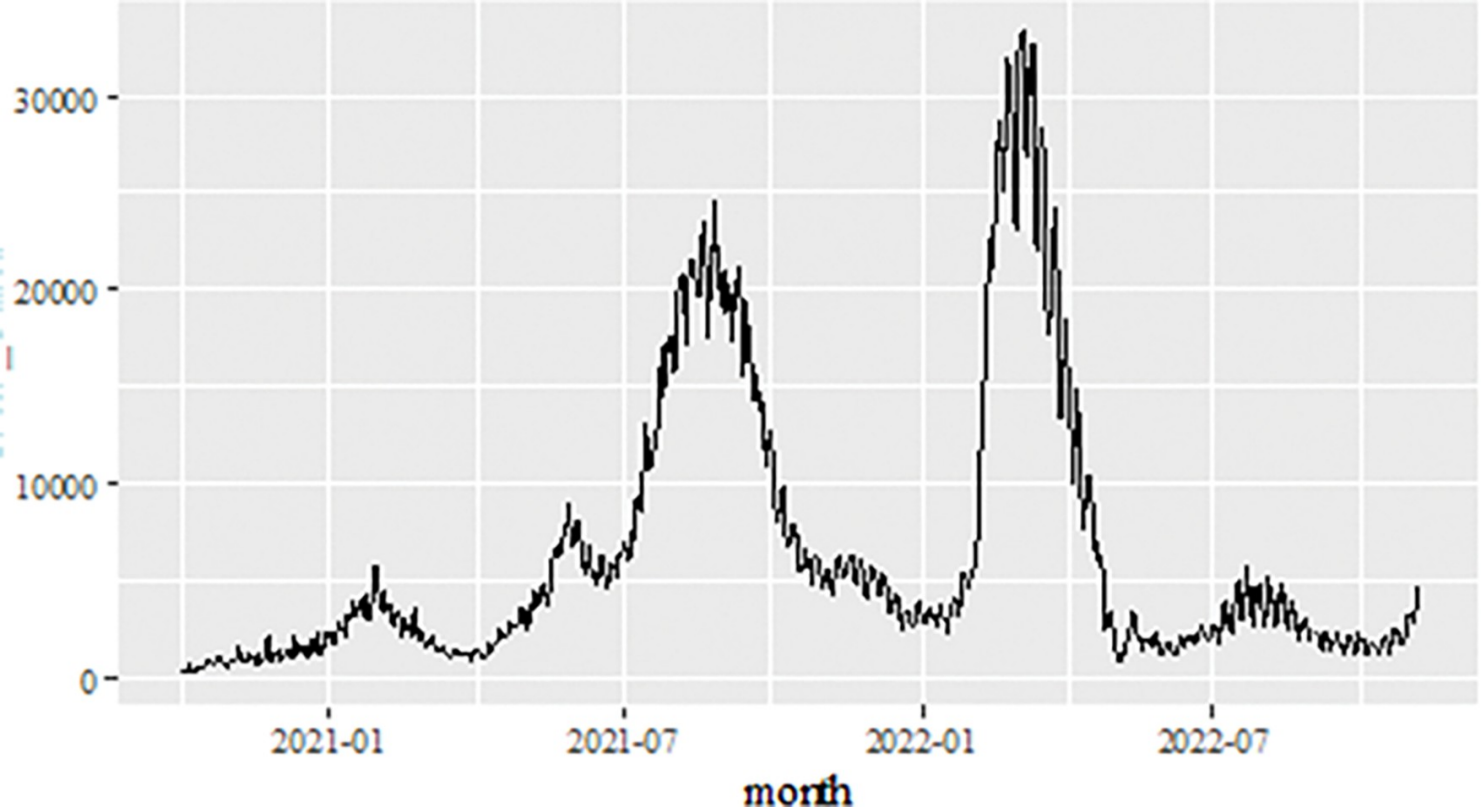
Malaysian daily new positive COVID-19 cases (1^st^ of October 2020 to 4^th^ of November 2022).

The daily new positive cases datasets series is recoded from the 1^st^ of October 2020 to 4^th^ of November 2022 (see Fig 2) contains 765 data points. The number of daily new positive cases of COVID-19 in Malaysia continued to show a significant increase starting in July 2021 dropped below 5,000 new cases. However, it’s continued an increased again around March-April 2022 to the maximum of 33,406.00. But this number showed a drastic decrease until November 4, 2022. The daily new positive cases of COVID-19 datasets, which is consider in this investigation and the COVID-19 datasets also have been extensively used with a vast variety of linear and nonlinear time series models including ARIMA, ANN and machine learning methods [8–10, 12, 14, 17, 20–26, 34]. The study of the daily new positive cases of COVID-19 has crucial as an indication of the effectiveness of preventive measures that have been, are being and will be taken by the authorities in controlling the spread of this epidemic more effectively.

Therefore, to investigate the performance of the proposal models on daily new positive cases of COVID-19 datasets, which is similar approach by Aisyah et al., [16] is used where the dataset is divided into two samples, known as training sample and testing sample. According to Aisyah et al., [16] and Nurul Hila et al., [17], the datasets should be divided into two (2) which are 70–80% the data for training and the remaining 20–30% for testing yields the greatest outcomes [36, 37]. The training data are used to assemble the models while testing data is used to evaluate based on the statistical measurement the forecasting performances of the models. Thus, in this study the daily new positive cases of COVID-19 data set are divided into two samples which the training data set and test data set. For training data sets consists of 612 observations from day 1 to day 612, which is 80% of the data sets from October 1^st^, 2020, to June 4^th^, 2022, exclusively used to formulate. The test sample data sets used about 153 observations from days 613–765 (20%) for the period of 5^th^ June 2022- 4^th^ November 2022 in order to evaluate the forecasting performance of proposed models.

**Fig 3:**
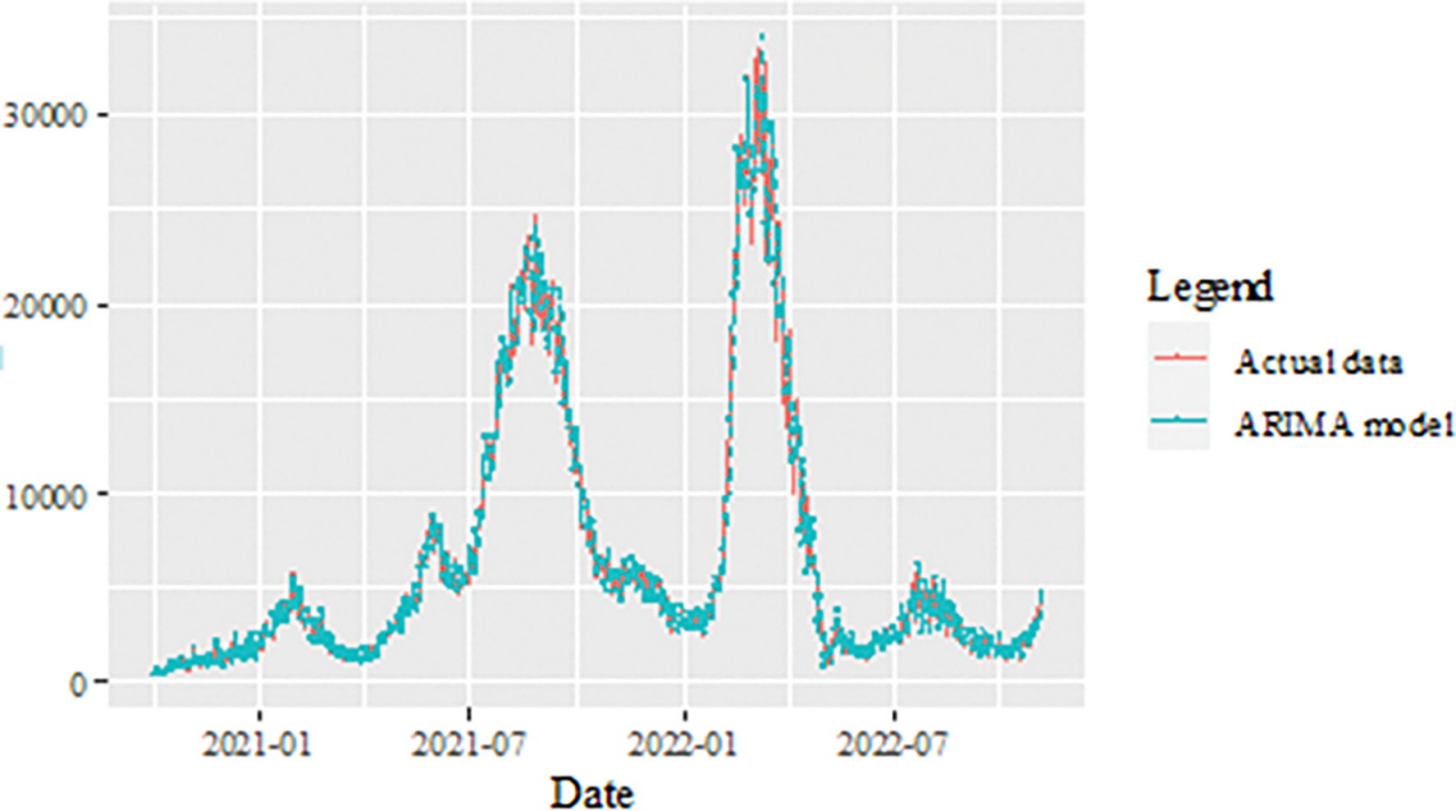
Results obtained from the proposed model for daily new positive COVID-19 cases dataset.

**Fig 4:**
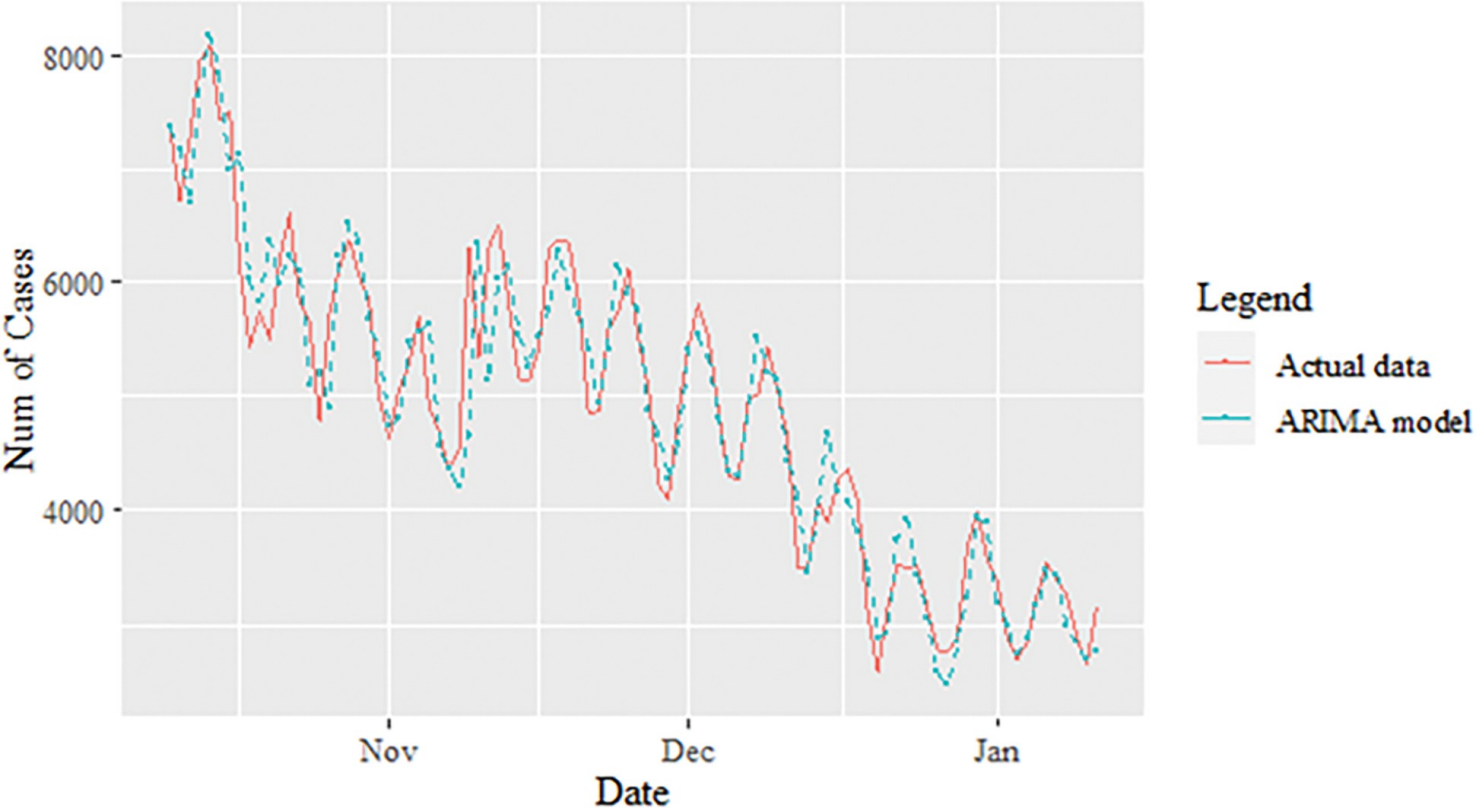
ARIMA model prediction of daily new positive COVID-19 cases dataset (test sample).

**Fig 5:**
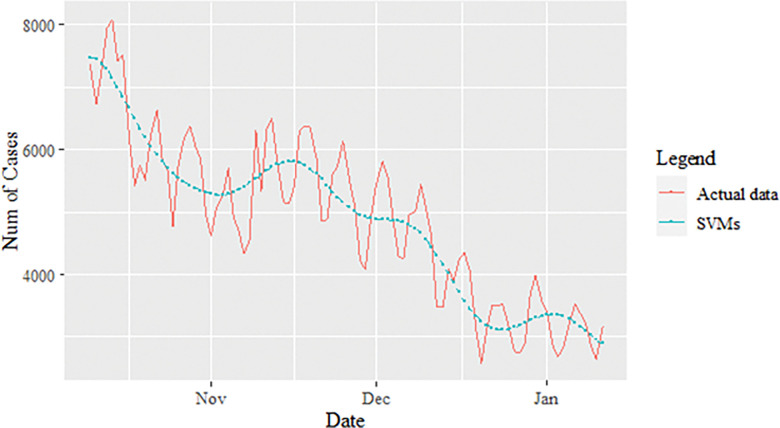
SVM model prediction of daily new positive COVID-19 cases dataset (test sample).

**Fig 6:**
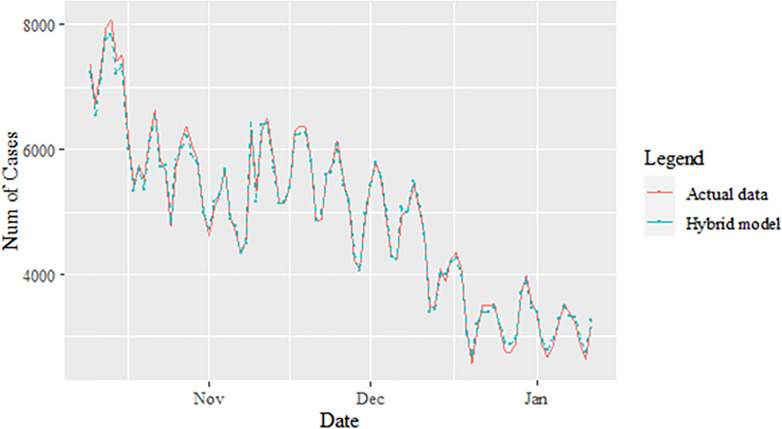
Proposed models prediction of daily new positive COVID-19 cases dataset (test sample).

**Fig 7:**
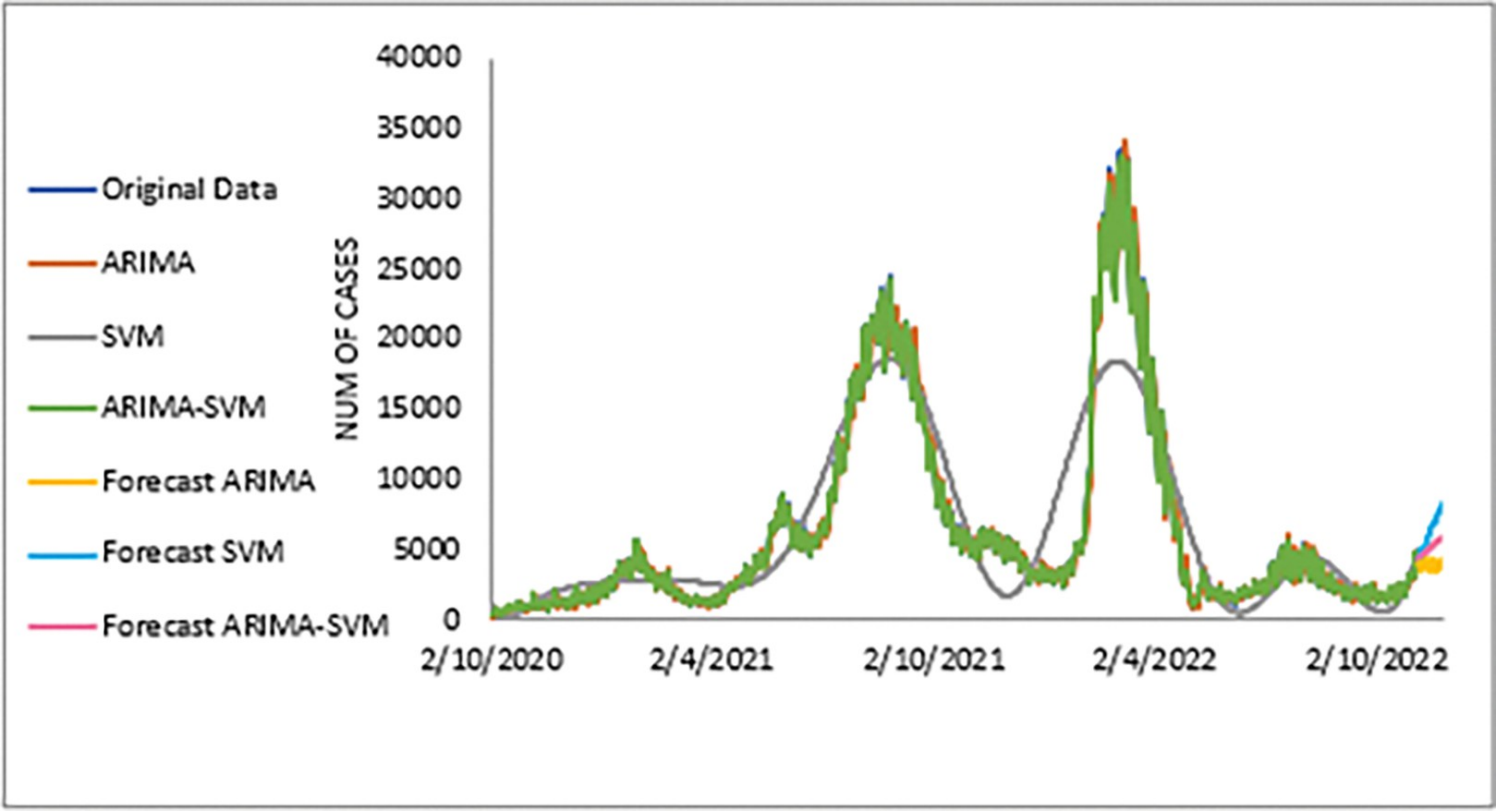
Actual and three weeks ahead forecasted values of ARIMA, SVM and ARIMA SVM models for new cases of COVID-19 of the 80% training and 20% testing set.

The performance of the proposed model of the daily new positive COVID-19 cases datasets are shown in Table 5. The results were obtained from the proposed models in terms of measurement error terms, namely MSE and MAE have the smaller values of 42552.7137 and 90.34845 Similar results were also obtained from the testing datasets with values of 61223.474, 0.05633, 247.4337 and 146.9841 for MSE, MAPE, RMSE and MAE, respectively. Based on these numerical results, the findings are examined in more detail using figures as illustrated in Fig 3. This figure, illustrates the estimated values for the proposed model (test sample) of daily new positive COVID-19 cases. As can be seen from this figure, the proposed model line closely matches the actual data. As a further example, Figs 4–6 provide estimated values of our model for test data and ARIMA, SVM, and SVM models for COVID-19 cases. A comparison of the proposed model’s lines for the test sample (Fig 6) with ARIMA and SVM models clearly shows that the proposed model’s lines are somewhat similar to actual data. Comparing the performance of our proposal models with that of ARIMA and SVM models, this indicated that our proposal models are efficient, accurate, and precise. In addition, as in Fig 7, the number of daily new positive COVID-19 cases is plotted. From this figure, the daily new positive cases of COVID-19 for Malaysia are forecasted for the forthcoming three weeks.

**Table 5:** Performance measures of the proposed model for daily new positive COVID-19 cases datasets.

Models	Train		Test			
	MSE	MAE	MSE	MAPE	RMSE	MAE
**ARIMA**	929843.169	611.0274	298988.28	0.15167	546.7982	397.57
**SVM**	8355184.483	2001.644	274588.16	0.15421	524.0116	390.3848
**ARIMA-SVM**	42552.7137	90.34845	61223.474	0.05633	247.4337	146.9841

Based on Table 6, we further analysed the performance of the proposed models for the daily newly positive COVID-19 cases dataset by comparing at the percentage of MSE, MAPE, RMSE and MAE. The study hypothesis investigates assumptions of the proposed hybrid model (ARIMA-SVM) approach to single ARIMA and SVM models. The proposed model achieved a higher percentage of improvement in MAE, MAPE, MSE and RMSE compared to the ARIMA model with improvements of 63.03%, 62.86%, 79.52%, 54.74%, where the parenthesis indicates the SVM model that results in (62.34%, 63.47%, 77.70%, 52.78%). Therefore, based on these results (Tables 4–6 and Figs 3–7), it can be concluded that the proposed model that has been developed has produced higher accuracy as well as efficiency compared to results achieved by ARIMA and SVM

**Table 6:** Percentage improvement of the proposed models with other forecasting models (The COVID-19 cases of daily new positive cases).

Model	MAE	MAPE	MSE	RMSE
**ARIMA**	63.0294	62.8602	79.5231	54.7486
**SVM**	62.3489	63.4719	77.7035	52.7809

### New deaths cases data forecasts

**Fig 8:**
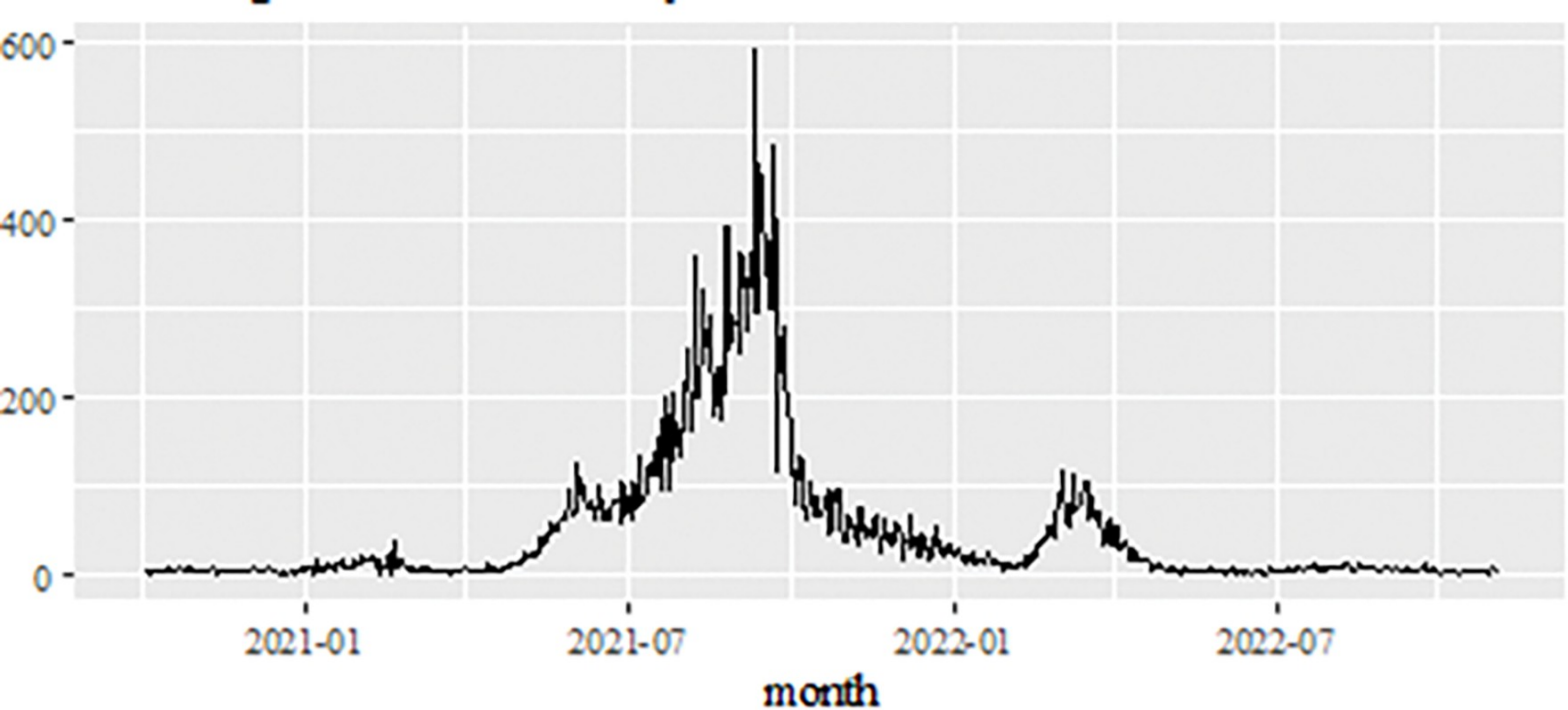
Malaysian daily new deaths COVID-19 cases (1^st^ of October 2020 to 4^th^ of November 2022).

Besides the Malaysian daily new positive COVID-19 cases datasets, the Malaysian daily new deaths cases datasets are also considered and used to analyse the performance of the proposed models. Similar to the daily new positive data set as well as the daily new death case data set, the recording period of this data set from 1^st^ of October 2020 to 4^th^ of November 2022 (see Fig 8) contains 765 data points and is divided into two samples. As a result of the increase in the number of daily positive cases of COVID-19 reported, this also shows that there is a significant increase in the number of deaths around 600. In order to formulate the model, the training data set involves 612 observations (80%) from October 1, 2020- June 4, 2022, the test sample uses approximately 153 observations (20%) for the period June 5, 2022- November 4, 2022, to evaluate the prediction performance of the proposed model.

A similar approach to the daily new positive cases of the COVID-19 dataset was used to study the performance of the proposed model on the daily new death cases of the COVID-19 dataset, the dataset was divided into two samples, namely, training sample and testing sample. For the training sample, it represents approximately 80% of the daily new death cases for the COVID-19 dataset (involving 612 observations with the period October 1, 2020, until June 4, 2022). The remaining 20% is for the test sample, involving approximately 153 observations starting from June 5, 2022- November 4, 2022.

The performance of the proposed models using the daily new deaths COVID-19 cases datasets is first characterized by statistical measurement such as the MSE, MAPE, RMSE and MAE as shown in Table 7. The results for the training data from this table show that the proposed model gives the smallest values of 49.4459 and 3.53812 for MSE and MAE values, respectively, compared to ARIMA and SVM for MSE and MAE values, respectively, compared to ARIMA and SVM. The same trend also occurs on the test data where all the values of the statistical measures used show the smallest values compared to the ARIMA and SVM models.

**Table 7:** Performance measures of the proposed model for daily new deaths COVID-19 cases datasets.

Models	Train		Test			
	MSE	MAE	MSE	MAPE	RMSE	MAE
**ARIMA**	697.999	11.8083	6.06741	0.56838	2.46321	1.92791
**SVM**	1409.19	21.8006	5.38920	0.53687	2.32146	1.85605
**ARIMA-SVM**	49.4459	3.53812	0.92630	0.19088	0.96303	0.76230

**Fig 9:**
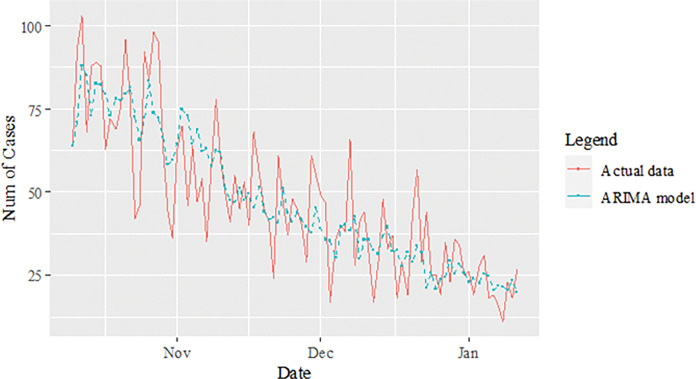
ARIMA model prediction of daily new deaths COVID-19 cases dataset (test sample).

**Fig 10:**
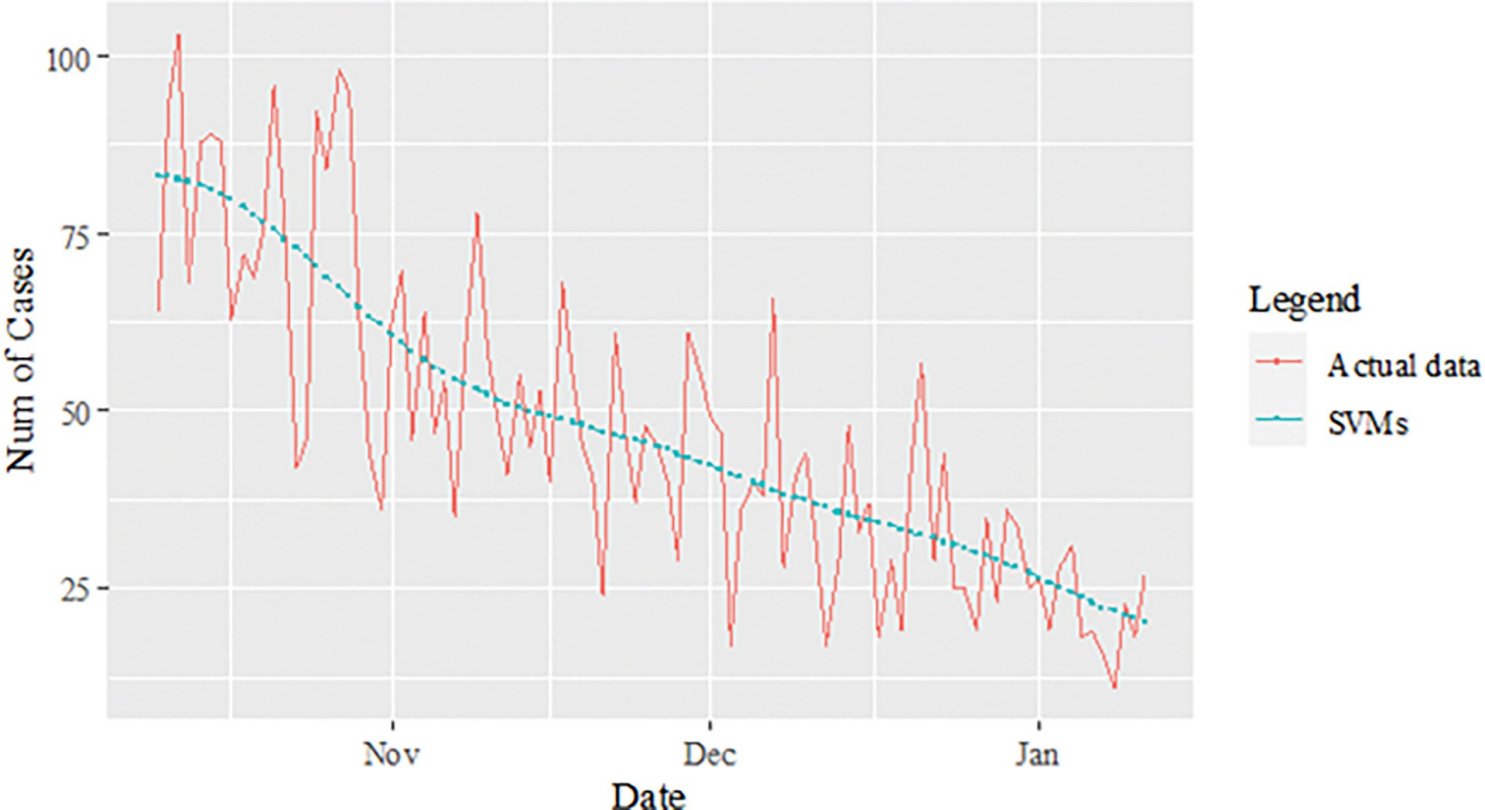
SVMs model prediction of daily new deaths COVID-19 cases dataset (test sample).

**Fig 11:**
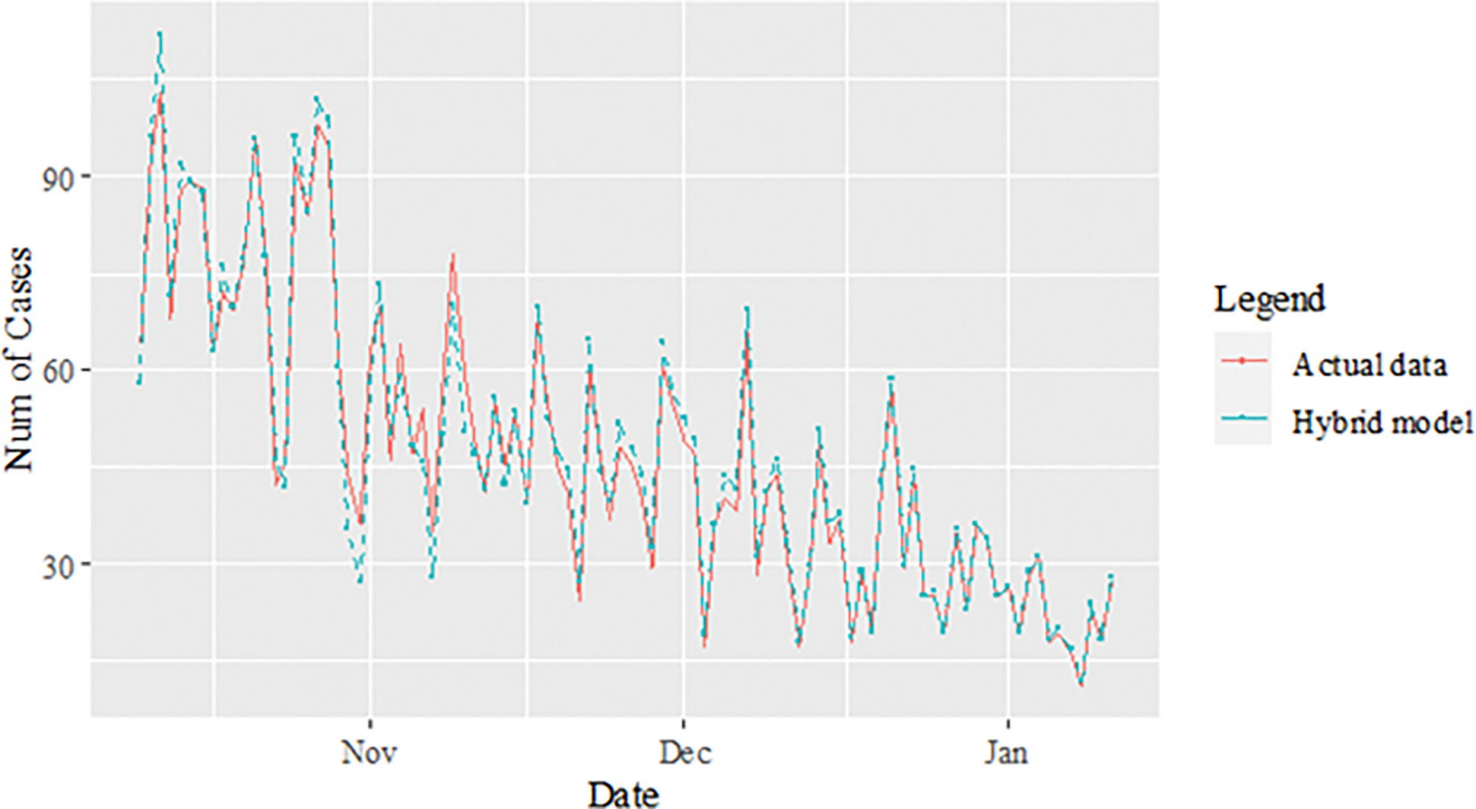
Proposed models prediction of daily new deaths COVID-19 cases dataset (test sample).

**Fig 12:**
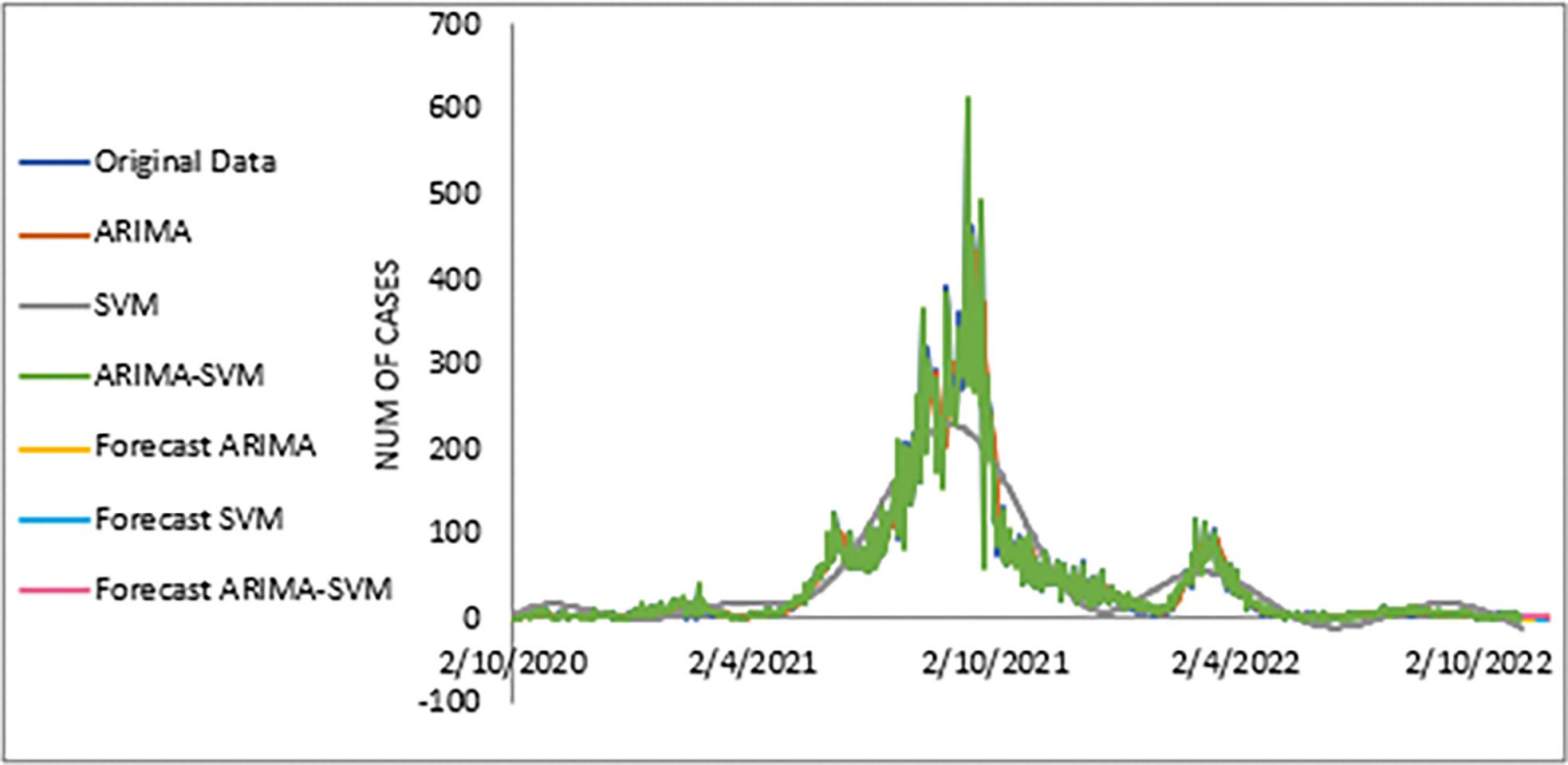
Actual and three weeks ahead forecasted values of ARIMA, SVM and ARIMA SVM models for daily new deaths COVID-19 cases of the 80% training and 20% testing set.

The study continues by investigating the estimated value of the proposed model for the daily new death COVID-19 case data set as illustrated in Fig 8. This figure clearly indicates that the proposed model line is almost no difference with the actual data. In addition, the estimated values of ARIMA, SVM and proposed models for test sample are plotted in Figs 9–11, respectively. Again, it clearly shows that our proposed model’s lines (Fig 12) for test sample are relatively closed to actual data compared to ARIMA and SVM models. This shows that the results of our proposed model are consistent with previous findings, which are efficient, accurate and precise compared to ARIMA and SVM models. In addition, as in Fig 12, the number of daily COVID-19 death cases is plotted. As a result of this figure, the daily new death cases of COVID-19 in Malaysia for the next three weeks are forecast to decrease, showing a downward trend in the next few weeks.

**Fig 13:**
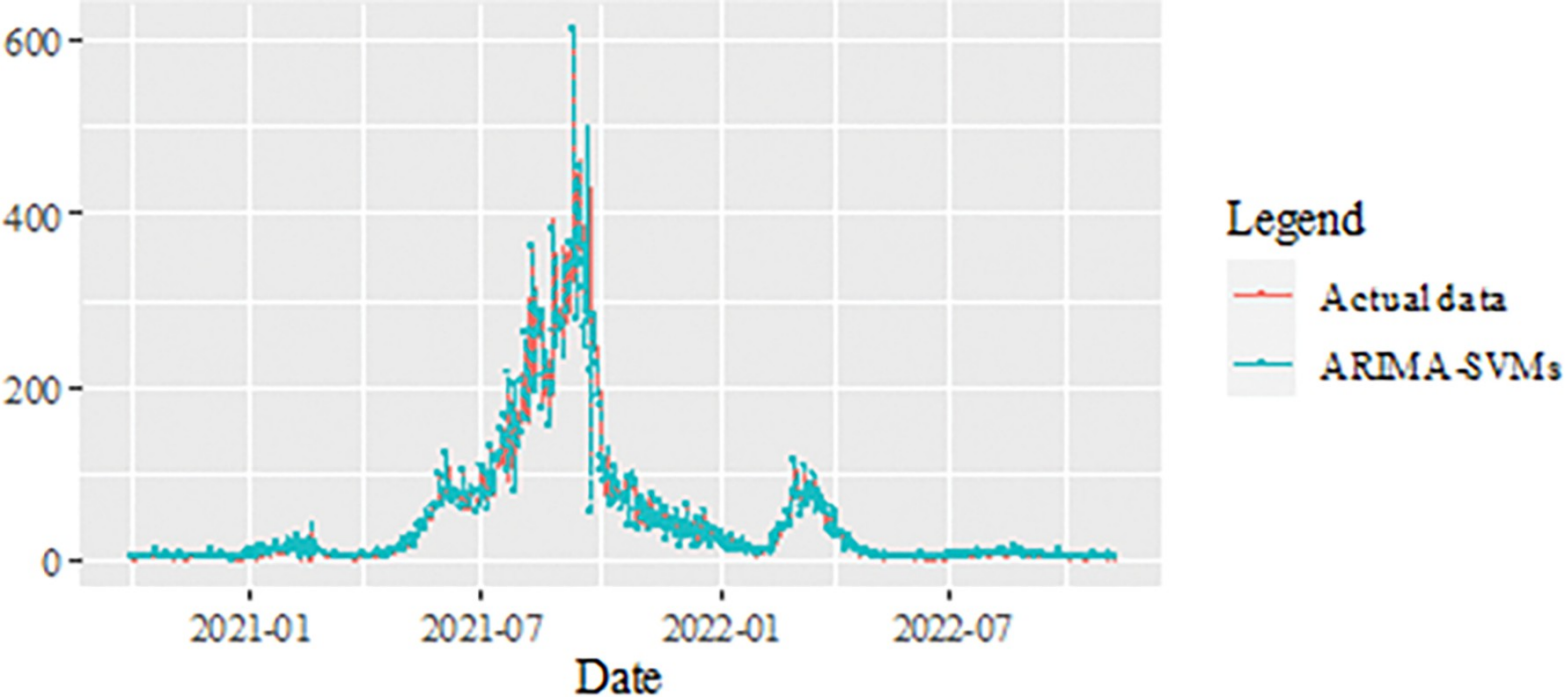
Proposed models prediction of daily new deaths COVID-19 cases dataset (test sample).

Here, a similar approach as in the daily new positive COVID-19 case dataset is used to investigate the performance of the proposed model for the daily new death COVID-19 case dataset through percentage MSE, MAPE, RMSE and MAE, as reported in the Table 8. Again, the percentage of improvement reveals that our proposed model produces better improvement for all statistical measures than the ARIMA and SVM models with results of 60.46%, 66.42%, 84.73%, 60.90%; improvement (58.93%, 64.45%, 82.81%, 58.52%) for MAE, MAPE, MSE and RMSE, respectively. The SVM model results reported in the parenthesis. The presented results (see Tables 7,8 and Figs 9–11, 13) clearly conclude that our proposed model has produced efficiently and accurately as well compared to ARIMA and ASV models.

**Table 8:** Percentage improvement of the proposed models with other forecasting models (The COVID-19 cases of daily new deaths cases).

Model	MAE	MAPE	MSE	RMSE
**ARIMA**	60.4598	66.4168	84.7332	60.9035
**SVM**	58.9289	64.4458	82.8119	58.5162

### New recovered cases data forecasts

**Fig 14:**
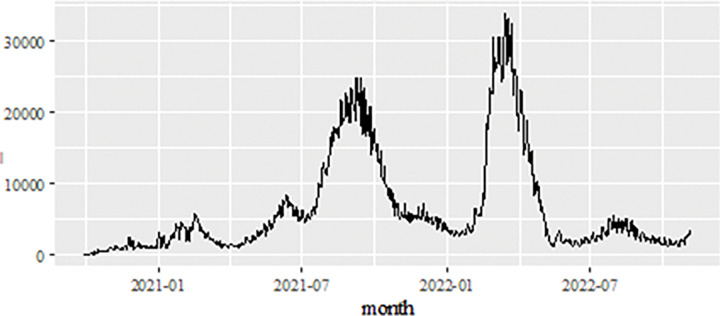
Malaysian daily new recovered COVID-19 cases (1^st^ of October 2020 to 5^th^ of November 2022).

The last dataset considered in this investigation to study the performance of the proposed model, is the dataset of new daily recovered cases of COVID-19 in Malaysia. Predicting Malaysia’s daily new recovered COVID-19 cases is equally important as the two datasets discussed earlier. The data used in this paper contain daily observation from the 1^st^ of October 2020 to 4^th^ of November 2022, giving 765 data points in the time series. The same trend is also shown by the number of patients recovered from COVID-19 where there is a significant increase twice. Starting in July 2021, the number of recovered patients also shows an exponential increase until it reaches over 22,500.00 in August 2021 (the time series plot is given in Fig 14) and drop. However, around March—April 2022, the number of recovered COVID-19 cases increased again until a maximum of 33,872.00 and then decreased and it showed a relatively stable movement after that. This dataset also divided into two samples, i.e., the training data set and test data set. Like the previous datasets, training data set is implemented in order to formulate the model, which involved 612 observations (80%) from 1^st^ October 2020-4^th^ October 2022. Whereas, to evaluate the forecasting performance of the proposed model, the test sample uses approximately 153 observations (20%) for the period 5 June 2022- November 2022.

Table 9 presented the performance of the proposed model of the daily new recovered COVID-19 cases datasets based on training sample and test sample. The results in Table 9 clearly show that the proposed training sample model produces the smallest MSE and MAE values with 99205.699 and 136.8519, respectively compared to the MSE and MAE models of the ARIMA model and the SVM model. For the test sample also revealed that the same scenario as the training sample ie, produced the smallest MSE, MAPE, RMSE and MAE with values of 26108.02, 0.0396, 161.5797 and 104.1002, respectively compared to ARIMA and SVM as well.

**Table 9:** Performance measures of the proposed model for daily new recovered COVID-19 cases datasets.

Models	Train		Test			
	MSE	MAE	MSE	MAPE	RMSE	MAE
**ARIMA**	1802678.36	804.4378	271462.22	0.1560	521.0203	387.2768
**SVM**	7636804.13	1890.917	239672.00	0.1504	489.5630	371.6573
**ARIMA-SVM**	99205.699	136.8519	26108.02	0.0396	161.5797	104.1002

**Fig 15:**
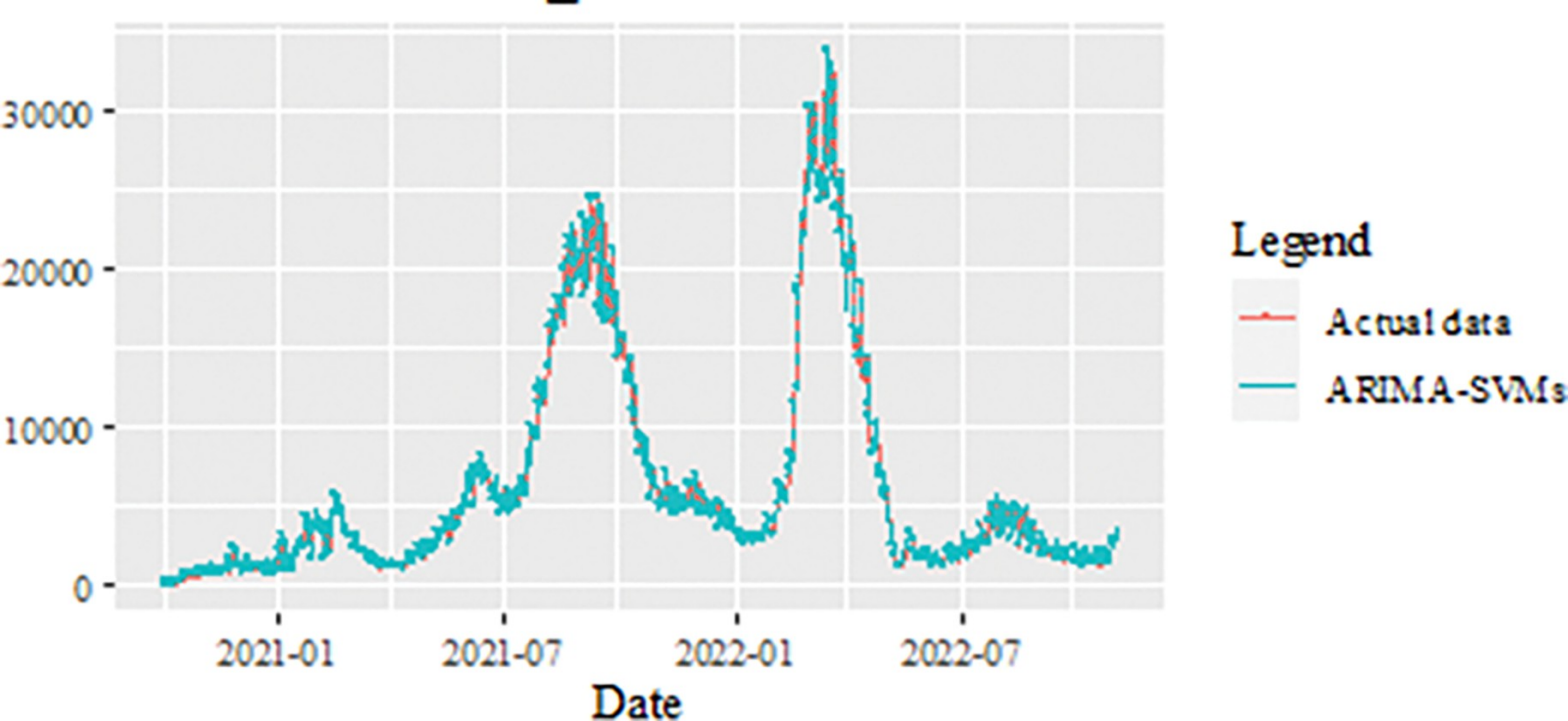
Results obtained from the proposed model for daily new recovered COVID-19 cases dataset.

**Fig 16:**
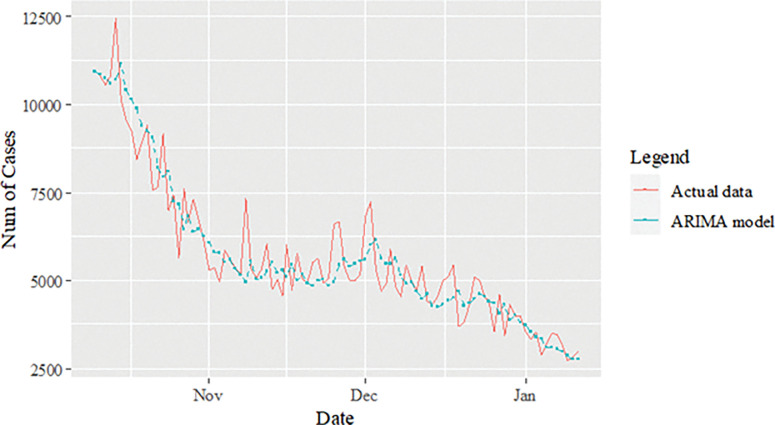
ARIMA model prediction of daily new recovered COVID-19 cases dataset (test sample).

**Fig 17:**
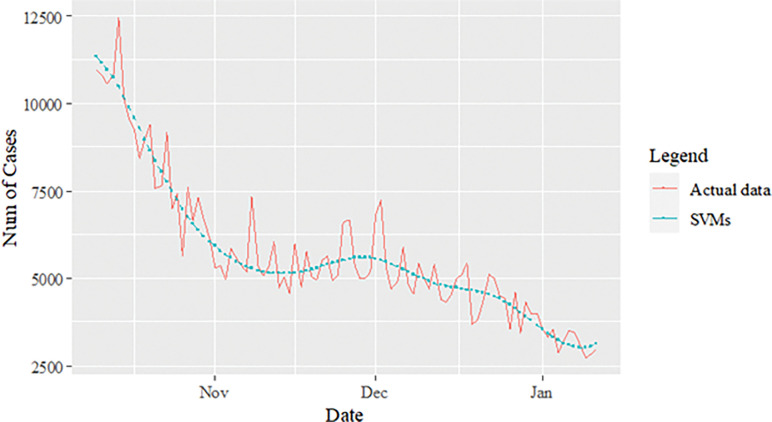
SVM model prediction of daily new recovered COVID-19 cases dataset (test sample).

**Fig 18:**
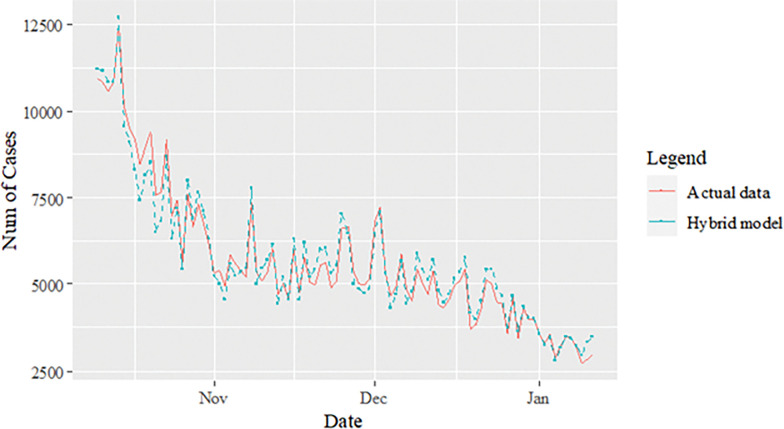
Proposed model prediction of daily new recovered COVID-19 cases dataset (test sample).

**Fig 19:**
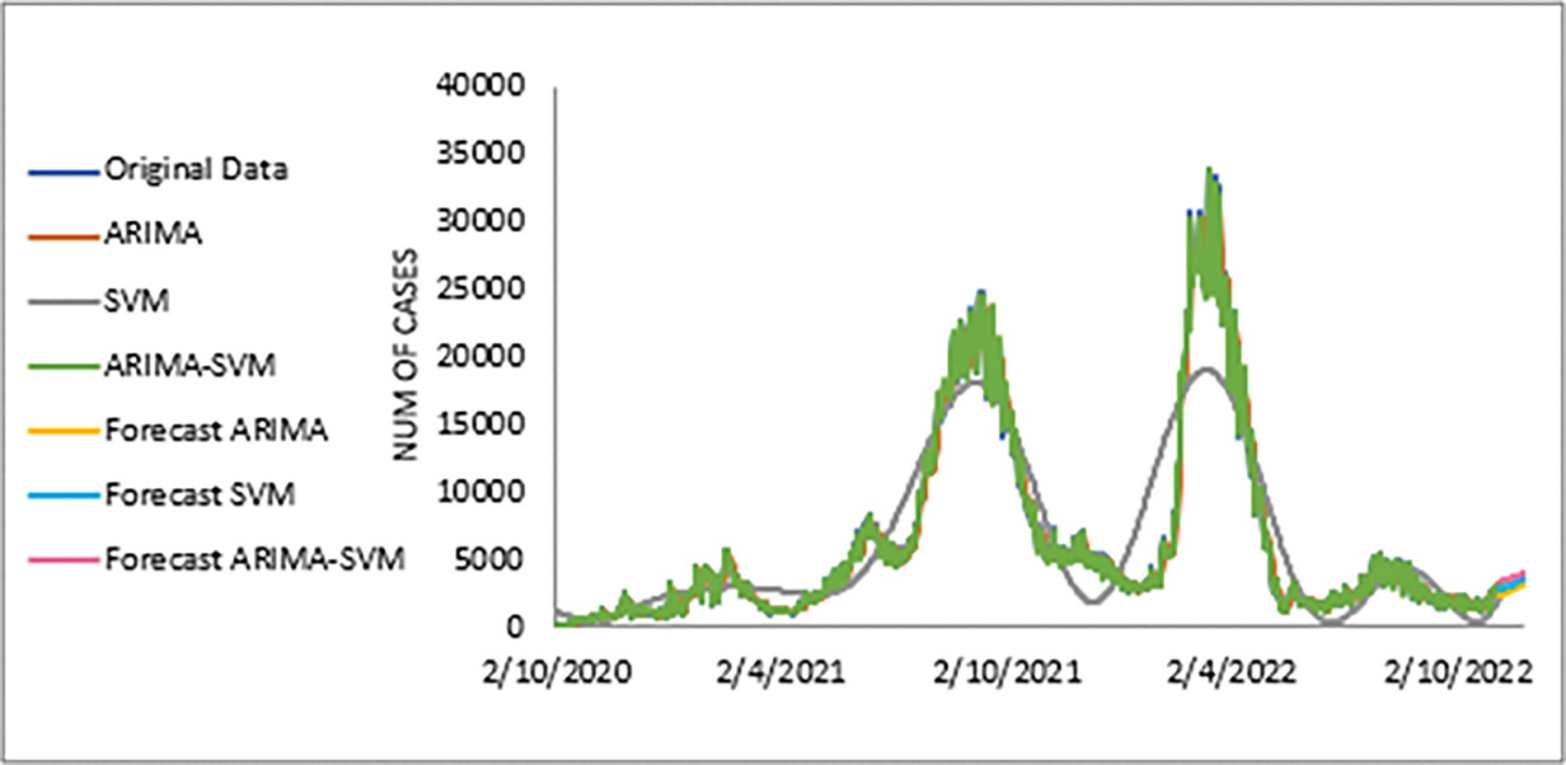
Actual and three weeks ahead forecasted values of ARIMA, SVM and ARIMA SVM models for daily new recovered COVID-19 cases of the 80% training and 20% testing set.

Meanwhile, the estimated value for the test sample of the proposed model for the dataset of daily new COVID-19 cases is depicted in Fig 15. Again, this figure clearly shows that the predicted value from the proposed models appear to be close to the actual values. A further investigation of the proposed model’s results is displayed in Figs 16–18. These three figures (Figs 16–18) reveal that the predicted values extracted from ARIMA, SVM, and the proposed model for the test samples seem to be close to the actual values. However, as we will see in Fig 8, these models are dominated by the proposed model i.e., they are closed to the true value. The number of daily new recovered COVID-19 cases is plotted as in Fig 19. In this figure, it’s clearly shown that the proposed model follows the original sharpness of the data. From this figure, the daily new recovered cases of COVID-19 for Malaysia are forecasted for the forthcoming three weeks and indicates that daily new recovered COVID-19 cases would increase in the upcoming days in Malaysia.

The performance of the proposed models for the daily new recovered COVID-19 cases datasets was further investigated for MSE, MAPE, RMSE and MAE in terms of the percentage, as reported in Table 10. By looking at the percentage of improvement for statistical measurements such as MSE, MAPE, RMSE and MAE, the results observed for the proposed model show a better improvement compared to ARIMA and SVM, respectively, with results of 73.12%, 74.62%, 90.38% and 68.99% improvement (71.99%, 73.67%, 89.11% and 66.99%) (where the results reported in the parenthesis are the SVM model). Therefore, based on the results, it can be concluded that the proposed model that has been developed has produced higher accuracy and efficiency compared to the results achieved by ARIMA and SVM models.

**Table 10:** Percentage improvement of the proposed models with other forecasting models (The COVID-19 cases of daily new recovered cases).

Model	MAE	MAPE	MSE	RMSE
ARIMA	73.1199	74.6153	90.3824	68.9878
SVM	71.9902	73.6702	89.1067	66.9951

## Conclusion

Accuracy and efficiency in predicting the spread of COVID-19 is crucial but often difficult for decision makers, especially the frontline and authorities. Although the spread of COVID-19 seems to be endless, but many efforts in the development of time series models, research to improve the effectiveness of forecasting models has never stopped. Among them is the hybrid approach and one of the most popular categories of hybrid models that decompose time series into linear and non-linear forms. in this study, a hybrid model as a combination of predictions produced by linear and some non-linear is proposed. The proposed model was investigated using three well-known COVID-19 data sets, namely, daily new positive cases, daily new death cases and daily new recovered cases based on (1) performance of the proposed model and (2) percentage improvement compared to ARIMA and SVM models. The proposed model with cross-validation check based on MSE, RMSE, MAE and MAPE is the most accurate prediction compared to ARIMA and SVM models. The performance of the proposed models produces the smallest values of MSE, RMSE, MAE and MAPE for both training and testing datasets. This means, the predicted value from the proposed model is closer to the actual value. In other words, the proposed model can generate estimated values more accurately and efficiently. In addition, percentage improvement of the proposed models against the ARIMA and SVM models (where the results reported in the parenthesis is SVM model) are 63.03%, 62.86%, 79.52%, 54.74% improvement, (62.34%, 63.47%, 77.70%, 52.78%); 60.46%, 66.42%, 84.73%, 60.90% improvement (58.93%, 64.45%, 82.81%, 58.52%) and 73.12%, 74.62%, 90.38% and 68.99% improvement (71.99%, 73.67%, 89.11% and 66.99%) for daily new positive cases, daily new deaths cases and daily new recovered cases, respectively. Therefore, our proposed models showed higher degree of precision and could be recommended for forecasting COVID-19. It can be concluded that the proposed model can be the best and effective way to improve the prediction accuracy performance, especially to predict and prevent the infection of COVID-19 cases is a priority.

## Limitations and future recommendation

An effort was made in this research study to forecast the total number of confirmed cases, fatalities, and recoveries of COVID-19 in Malaysia. Nowadays, the change in daily numbers of COVID-19 is affected by a very large number of factors, such as the population’s adherence to prevention measures, vaccination, social isolation, and new variants of the virus. As such, in order to improve future predictions and forecasts, it is imperative that the study of COVID-19 be taken into consideration in terms of (i) the clinical and behavioural aspects, and (ii) the possibility of underreporting cases, deaths, or delays in notifying as part of the study of COVID-19 in the future. Besides that, to improve the accuracy of the forecast in future work, investigation in SVM performance with different kernel functions and optimal hyper parameters of SVM forecasting model can be developed. Next, multi-step forecasts can be centralized in the future work since only one-step- ahead forecasting is considered in this paper. It is proven that multi-step forecasts can make the trading system much more realistic [38]. Finally, another approach, such as bootstrapping, can also be added as a hybridization of ARIMA and SVM [39]. Bootstrap is a reliable method given the lack of researchers adding this method in daily cases of COVID-19 forecasting. Many studies have shown that the bootstrap resampling technique provides a more accurate estimation [17, 40–42].

## Supporting information

S1 Dataset (XLSX)
